# Negotiating dignity in public geography: The ethics of public engagement in pandemic times

**DOI:** 10.1111/area.12818

**Published:** 2022-07-20

**Authors:** Anna Plyushteva

**Affiliations:** ^1^ School of Geography and the Environment University of Oxford Oxford UK

**Keywords:** COVID‐19, dignity, essential workers, ethics, human geography, public engagement

## Abstract

In this paper, I reflect on some of the ethical dimensions of public engagement with geographic research. The paper draws on my recent experience of a project entitled ‘Not working from home’, which sought to make visible the everyday experiences of essential workers during the COVID‐19 pandemic. The project was intended as a space for essential workers to document their daily lives using text, images and video, enabling them to engage with each other, while also informing the wider public about the everyday challenges of not working from home during the pandemic. The paper discusses some of the ethical implications and challenges of conducting this project, drawing on a critical engagement with dignity as an ethical framework for public engagement. I discuss the implications of calling workers ‘essential’, the role of collective and professional identities explored by the participants, and the impact of offering rewards. I also ask some broader questions on the role that the concept of dignity might play in the ethics of public engagement with research in human geography.

## Short Abstract

Dignity is a widely used concept and one with a deeply problematic history. In different ways, it is already being used in geographic research. This paper uses the example of the Not working from home project to discuss what a relational understanding of the concept of dignity can bring to the ethics of public engagement with research in geography.

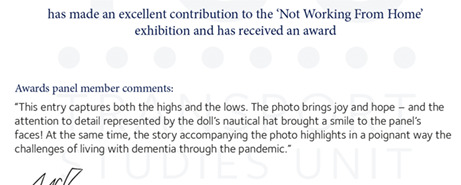

## 1 INTRODUCTION

In this paper, I reflect on some ethical dimensions of public engagement with geographic research. The discussion draws on a recent project entitled ‘Not working from home’, which sought to make visible the everyday experiences of essential workers during the COVID‐19 pandemic. While the notion of public engagement with research has traditionally referred to educating ‘the public’, or sharing out the benefits of research, the interconnections between public engagement, research and their different publics are now recognised as more complex and multidirectional, potentially resulting in harmful as well as beneficial interactions (Esmene, 2021; Smith, 2013). In ‘Not working from home’, the aim was to create spaces for essential workers to engage with each other and with different publics, with research playing a secondary role. While I *felt* strongly that I wanted to embark on this project (I discuss the role of the affective dimension below), it was important not to construe it as innately beneficial or uncontroversial. An obvious example of the ethical challenges involved concerns using the term ‘essential worker’, a category widely debated during the COVID‐19 pandemic, and not always with input from the workers themselves.

In the discussion that follows, I interrogate some of the ethical decisions I made, considering them in dialogue with recent work on the role of the concept of dignity in geography (Grossmann & Trubina, 2021, 2022). I have three reasons to focus on dignity. First, given the growing attention geography departments in the UK are placing on ‘impact’ (Hammett et al., 2019), there is a need to examine the specific ethics of public engagement, and a dignity framework is one way of doing this. Second, due to its long history of codification in other disciplines such as medicine, as well as the deeply problematic ways its claims to universality have (not) been put into practice (Clark Miller, 2017; Nahman, 2008), dignity opens up a particular set of questions around doing geography ethically. These are related to, but distinctive from, the approaches associated with, for instance, care ethics; (Wood & Swanson, 2020). Finally, the ubiquity and relative taken‐for‐grantedness of references to dignity in institutional ethical approval processes suggest that such a discussion may be overdue. At the same time, this paper does not aspire to resolve the many questions which result from bringing a ‘big’ concept such as dignity to bear on public engagement with human geography. Instead, by critically considering the minutia of ethical choices made, I aim to illustrate some of the utility and limits of thinking with dignity.

## 2 PUBLIC ENGAGEMENT WITH GEOGRAPHIC RESEARCH

A growing body of reflexive and critical work has shed light on the different ways in which geographers engage with publics beyond the academy (see Fuller, 2008; Saunders & Moles, 2013). Increasingly, such accounts have questioned the framing of public engagement as an intrinsically positive societal contribution, and have unpacked the relations of power and exploitation which can define such initiatives (Esmene, 2021). These critical conversations have enabled reflections on the specific, situated practices which geographers engage in when doing public engagement work. This paper seeks to extend such critical reflections, particularly in light of the many harms inflicted by the COVID‐19 pandemic and its far‐reaching consequences for research and education. On one hand, both before and during the pandemic, public engagement projects have been evaluated mostly in terms of quantitative measures of greater or lesser ‘impact’, rather than their ethics or politics. On the other hand, more and more critical scholars have asked uncomfortable questions about the true impact on ‘engaged’ publics (Darby, 2017). As the ‘Not working from home’ project was not only initiated during the COVID‐19 pandemic, but also entailed a public conversation about its consequences, these questions seemed especially pertinent.

## 3 PANDEMIC ETHICS AND ENGAGING WITH DIGNITY

While the literature on pandemic ethics is substantial, it is almost exclusively concerned with the ethical implications of pandemic measures, such as implementing restrictions or allocating scarce medical resources among different vulnerable populations (DeBruin et al., 2012; McGuire et al., 2020; Thomas et al., 2007). A few studies have engaged with the ethical implications of conducting social science research during the COVID‐19 pandemic, but not those of public engagement specifically (Buckle, 2021). This is significant not only because engagement for purposes other than data collection is a distinctive activity. Importantly, while primary data collection in the midst of COVID‐19 could have been justified through its utility to pandemic management and decision‐making, the ‘justifiability’ of a public engagement project is not straightforward (Hammett et al., 2019). The pandemic conditions and the disciplinary context outlined in the Introduction encouraged me to turn to a concept widely used in the medical sciences and social care, that of dignity.

The concept of dignity is central to the statutes and practices of international human rights law, which is itself the basis of ethical principles and procedures for much social science research (Becker, 2021). At the same time, dignity does not have a universally accepted definition, and has often been criticised for its vagueness and internal contradictions (Forst, 2011; Jacobson, 2007). Social science research which draws on the notion of dignity has variously focused on dimensions such as respect, self‐respect, recognition, belonging and self‐determination, although this list is not exhaustive (Gupta, 2006; Grossmann & Trubina, 2022). Broadly, dignity can be defined in terms of the moral status of a person, their worthiness of respect (Misztal, 2013). The influence of the concept is far‐reaching; innumerable legal documents posit dignity as inherent, inalienable and equal for every human being, the fundamental basis for the universal human rights framework. However, a rhetoric of dignity has often been shown to do little to counter injustice. As Clark Miller points out, dignity is ‘both a highly influential and a profoundly problematic concept’ (Clark Miller, 2017, p. 108). Thus, postcolonial and feminist scholars have documented the impacts of narrow, hierarchical and Eurocentric definitions of rationality, posited on the basis of Kantian ethics as that which makes one worthy of human dignity. Supposed to bestow and protect universal rights, such approaches have often resulted instead in the justification of violence and exclusion on the basis of race, gender, disability and class (Clark Miller, 2017; Fanon, 2008).

Despite this problematic history, dignity has acquired new and more hopeful meanings in both theory and practice. Political activists from the Movement for Black Lives and the Euromaidan protesters in Ukraine, among others, have drawn on a vocabulary of dignity in formulating their demands (Grossmann & Trubina, 2022). More recent academic engagements have called for relational, affective and situated understandings of dignity, conceptualisations which speak closely to contemporary theoretical concerns within human geography (Clark Miller, 2017; Grossmann & Trubina, 2022). These ideas build on, and extend, earlier work on social dignity; that is, dignity as a contextual and contingent arrangement which is affirmed or eroded in interpersonal relations (Jacobson, 2007). Relational approaches differ from the idea of social dignity in a range of ways, but two should be highlighted in particular. First, a relational view does not locate dignity within individuals, or frame it as contingent on hierarchical notions of rank, status, or ‘dignified’ social standing. Second, relational conceptualisations pay close attention to affect and emotions (in contrast to the view of rationality as the opposite of emotion and the true basis for dignity).

Drawing on a situated, relational and affective understanding of dignity, I explore the negotiation of dignity in the ‘Not working from home’ project by locating it within specific interactions, the result of which is *felt* by those involved. Importantly, these interactions are not cumulative in any straightforward sense; that is, they do not add up to a project which either promotes or erodes participants' innate dignity. As I aim to demonstrate, while public engagement projects require explicit ethical commitments, the latter cannot be meaningfully or sufficiently expressed in terms of one individual ‘affirming’ another's dignity by carefully choosing some actions over others. Instead, I explore an explicitly geographical, spatially and temporally contingent way of approaching dignity which is preliminary, partial and in need of continuous reworking, but one which begins to articulate a basis for ethical obligations to participants.

## 4 ‘NOT WORKING FROM HOME’ AND RECOGNISING ESSENTIAL WORKERS

The ‘Not working from home’ project sought contributions from essential workers for an online exhibition of photos, short video recordings and text (available at https://www.notworkingfromhome.org/). The exhibition was to document everyday life during the pandemic for those who continued to have to go to a workplace outside of the home. My original intention for the project was that it would focus on life outside of the workplace (e.g. the commute, shopping, leisure), in order to avoid the potential ethical and practical pitfalls of participants taking photos and videos at work, and also to provide participants with what I assumed to be much‐needed respite. However, many of the contributors chose to submit stories from their place of work, a point I discuss further below.

The project built on my long‐term research interests in everyday mobilities and immobilities. In many ways, however, the project was an emotional undertaking, something I felt the need to do, rather than a logical next step in a planned academic path (Askins, 2009). Since March 2020, I had felt both relief and guilt about being able to work from home. I had also felt much frustration: it seemed to me that the public focus, especially in the earlier stages of the pandemic, was disproportionately on the challenges and adaptations associated with a shift to home working. The particular fears, uncertainties and even financial penalties of continuing to go to a workplace had received proportionately less attention (see, for instance, Rose‐Redwood et al., 2020; Plyushteva, 2022). By creating a space for essential workers to share first‐person accounts of their everyday experiences of going to work, the project hoped to invite recognition of their difficulties and anxieties, helping valorise particular kinds of pandemic work in a way that was more nuanced and cautious than the prevalent rhetoric of ‘heroism’ (see discussion below).

Between March and June 2021, the project gathered 70 stories from across different sectors and locations (mostly in the UK). The need to minimise social contacts during the Covid‐19 pandemic meant that all interactions with participants—inviting essential workers' stories; receiving and publishing them; discussing and publicising them; asking for feedback; and conducting follow‐up interviews with participants, took place online. Conducting public engagement entirely online raises a number of particular ethical questions, not least in the way it excludes some, while playing into the perceived, and sometimes harmful, indispensability of digital interaction for others (see Fuller & Askins, 2010 for a discussion). In late 2021, I began conducting follow‐up research interviews with those essential workers who had opted in; these are ongoing at the time of writing.

The use of ‘essential’ in a project designed to foster a particular mode of recognition of a group of people which the project author does not belong to has important ethical implications. These were reflected in both academic publications and newspaper columns, as the notion of ‘key’ or ‘essential’ work became pervasive in public conversations across the world almost overnight (Sainato, 2020). Although the very use of the word in itself may imply recognition, the specific circumstances and practices through which recognition is evoked, matter. As a minority of voices have pointed out, the notion of ‘essential’ came to carry an increasingly unsubtle element of coercion (Orleck, 2020). It came to designate being expected to continue to leave home and go to work; in other words, to do that which was considered too dangerous for most others during a pandemic, often despite low pay, inadequate protection and insufficient information on the risks involved. As some project participants confirmed, public celebrations only added to a sense of unfreedom and imposed expectation of self‐sacrifice. During 2020, public celebrations of essential work had become commonplace. While initiatives such as the weekly doorstep applause known as ‘Clap for Carers’ generated a sense of togetherness and even empowerment for those doing the clapping, others including workers themselves took to national and social media to express their unease (Wood & Skeggs, 2020). Furthermore, several project participants felt that celebrations created and entrenched various hierarchies within the ‘essential workers’ category, with healthcare workers recognised and many others, from railway engineers to school cooks, largely invisible. It was thus important that the project avoid becoming a ‘celebration’ of essential work along similar lines.

However, these considerations had to be weighed against the practical advantages of using the term ‘essential’. Its adoption enabled the key public of the project to identify themselves quickly and easily, hopefully reaching as many people as possible. Ultimately, a public engagement project cannot happen unless someone engages with it, and the world of digital communications is a crowded one. Similarly, despite my own reservations, it was important that the project did not become a platform for my own views, thus silencing the range of opinions workers themselves held in relation to being called ‘essential’. Using, for instance, a list of occupations would have always been partial, and thus limited some potential participants' ability to determine their belonging in the project's publics. By contrast, using ‘essential’ came with its own set of exclusions. Ultimately, with both options unsatisfactory, the decisive factor was my own positionality in relation to the group being described. As an unmistakably non‐essential worker, working from home with very little difficulty or exposure to the COVID‐19 virus, not calling workers essential would have carried greater ethical significance than doing so. However, the project needed spaces in which participants could subvert some of the dynamics around the naming and celebration of essential workers, while maintaining an open‐ended mode of recognition. Whether participants wanted to challenge the rhetoric of essential work, to ‘complain’ about everyday tasks, or indeed to embrace the narrative of the public's gratitude, the project needed to find ways to allow for these stories to co‐exist. These aspirations reflected the conceptual tensions of viewing dignity as collective rather than individual, but still requiring an open‐ended and non‐scalar view of the collectivities involved.

I aimed to speak to these concerns in several different ways. First, the project website prominently featured different occupations in turn, aiming not to prioritise particular sectors. Additionally, careful consideration was given to the project's name. Designed to be understated and mundane, the title of ‘Not working from home’ sought to emphasise just that—that essential work involved a particular kind of daily routine outside the home. Thus, the relations which the title sought to emphasise were those of particular spatialities and im/mobilities, rather than of occupations or job titles. The routine of going to work, previously the familiar norm for so many, had suddenly become alien and invisible to those locked down at home (including myself). Thus, the project title invited conversation and reflection around the previously ordinary, both familiar and unknown, aspects of essential workers' daily lives, such as commuting, paying bills, getting coffee and making small talk in the workplace. As the next section shows, this open‐endedness proved to be key, as many of the participants' plans for the project turned out to be different to my own.

## 5 LOCATING PARTICIPANTS' STORIES

As discussed above, I thought that ‘Not working from home’ would mostly be a space for participants to explore various ‘non‐heroic’ aspects of daily life. It quickly became clear that the majority of participants wanted to contribute stories which foregrounded their professional identities. By the end of the project, 44 of the 70 stories submitted were about work, such as the nature of the job itself and relationships with co‐workers, and the changes both had undergone during the COVID‐19 pandemic. Several participants made clear in follow‐up interviews that making prominent their professional identities was important to them. Beyond the debate concerning the ‘essential’ nature of their work was a whole host of professional challenges, achievements and experiences participants wanted to share. One participant, a childcare worker, documented their reaction the first time they were handed a face mask to wear in the workplace; another care worker described the painstaking efforts involved in converting a care home's traditional Easter Egg Roll into an indoor activity. These accounts echo recent publications on the challenges, interactions, kindness and fears essential workers have shared (see, for example, Veazey et al., 2021). They also reflect a longstanding focus on ideas of belonging in the literature on dignity (Grossmann & Trubina, 2022). Unlike conceptualisations of belonging around geographically bounded communities or enduring identities, belonging here took the form of fleeting and indeterminate moments of connection. Using social media channels, participants often responded with warmth and empathy to the published stories of others. These interactions enabled participants to explore shared identities, to joke, complain or reminisce, in the process dislocating the ‘static’ and individualised recognition of the project website with it list of submissions. They hinted at collective and relational notions of dignity constructed around the range of types of work represented in the project, both distinctive and with many experiences of everyday life, particularly not being at home, in common. All of this is not to suggest that these social‐media‐based interactions were necessarily straightforward or beneficial for those engaging in them. The performance of individual and collective identities through social media is inevitably a complex and contingent process (Lee, 2020). While not free of uneven power relations, contradictions, or tensions, the social media interactions nonetheless helped decentre the project website as the place where participants were to exhibit their stories. These additional spaces enabled those participating to add to their story, or respond to feedback to it, and to do so in often more informal and spontaneous ways. However, as several participants successfully wrestled the ‘Not working from home’ narrative away from the project itself, taking ownership of what they wanted to say about themselves in public in this context, so the project ‘pushed back’, as I tried to negotiate relinquishing control with the academic pressures I myself faced. ‘Doing’ public engagement as an academic geographer comes with its own objectives and agendas, related to career progression, outputs, securing funding and stable employment (Esmene, 2021). As participants shared moving and poignant stories with each other on the project's Facebook page, I agonised over whether, and how, to encourage them to submit these stories also on the project website, adding to its ‘impact’. About halfway through the project, I decided to stop posting such messages of encouragement. I reduced my interference in these social media conversations to a minimum, focusing instead on sharing and promoting the stories submitted to the project website.

## 6 PARTICIPANT REWARDS

Another significant ethical challenge concerned the idea of giving awards to some of the participants. This seemed necessary in order to publicise the project and recruit as many participants as possible. Similarly, awards seemed an appropriate way of expressing gratitude—for investing time and effort into participating, and for doing difficult and risky work. At the same time, only a small number of awards could be made available on the project's budget, which meant participation would have a competitive element. A prize‐draw or lottery was discounted, being in itself exclusionary. However, the implications of creating any form of competition among participants created a deep sense of discomfort.

**FIGURE 1:**
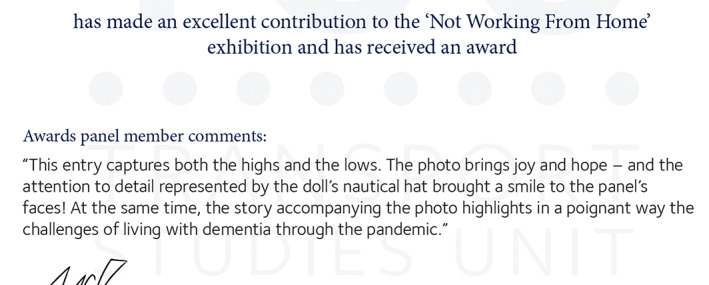
Example of awards certificate

The sense of unease was amplified in cases where participants' stories focused on their own and their families' financial struggles. These stories made me feel angry and upset, even if the poor remuneration of work such as care or delivery is well known and well documented (McDowell et al., 2008). It was understandable why it was especially tempting, then, to prioritise rewarding the authors of those stories. However, this was problematic. First, as poverty and financial struggles often come with shame and stigma (Grossmann & Trubina, 2021), participants who had not mentioned acute money problems could not be assumed to not have them. Similarly, I did not know how participants who had discussed their financial difficulties be made to feel by the award. Whether in the context of material deprivation participants experienced awards as charity, recognition, respite, or support is a question which speaks directly to longstanding concerns in the dignity literature (Grossmann & Trubina, 2022). Ultimately, the decision was made to create as many layers of separation as possible between the core project activities, and the awards process. A first key step was to minimise my own involvement in making awards decisions. I asked a different person each month (several awards were allocated monthly over the course of 4 months) to choose the recipients for that period. A diverse group of academics with interests in visual methods and representatives of the creative arts were invited to select the winning contributions. They were deliberately not presented with specific criteria to apply in making their decisions. They could choose their favourites using any criteria they wanted, as long as they were able to summarise the reasons for their choice in a few sentences, and communicate it to the recipient in the form of a direct quote on an award certificate. The comments were not to centre the positive or negative attributes of any submission. This aimed to re‐frame the awards process—an inherently unequal, institutionalised and fraught arrangement, as a relatively open‐ended human interaction in which one person could, in their own words, express gratitude, appreciation and respect to another. An example is included in Figure 1.

These comments of the awards panel were also published on the project website for all participants to read. Comparisons between the stories, awarded or not, were further decentred by ensuring each submission received equal coverage in the social media channels of the project. On the project website, award announcements were separated from the main project gallery, where ‘winners’ were not indicated.

Despite this, framing some contributions as being better than others remained a source of unease. Negotiating dignity within processes of selection, competition and reward illuminated the project's limits in stark terms. On one hand, some of the 10 participants who were recognised with awards spoke in follow‐up questionnaires and interviews of a sense of being seen and appreciated. One felt the award reminded them of their individual role in a significant moment in world history. The most tangible benefit of giving awards was the way in which some of the recipients acquired a stronger voice as a result. Thus, two recipients became very active on the project's social media pages, reacting to the submissions of others, encouraging them, and inviting colleagues to take part. Another recipient shared a detailed social media post about the joy brought by the award. Thus, in some cases, awards served as feedback, confirming to participants that their stories were valuable, valued and seen. This was an important message to send as part of a project which otherwise placed great emphasis on recording supposedly mundane experiences of everyday life. And yet, on balance, the ethical implications of competition and awards in a public pandemic geography may be too profound to be compatible with a focus on dignity. A similar project in the future could ask participants themselves to select award recipients, or recognise the time invested in the project in other ways.

## 7 CONCLUSION

As all ethical relations, navigating dignity as part of ‘Not working from home’ was replete with contradictions, ambiguities and often unease (Nuhrat, 2020). In describing the seemingly mundane decisions that were made around the title of the project and its organisation, I have aimed to think through the practical negotiation of dignity public engagement entails. Working on the ‘Not working from home’ project has also prompted further reflections on the range of responses to the COVID‐19 pandemic from academia in general, and geography in particular (Hall, 2021). As the literature on the medical ethics of pandemics demonstrate, there is limited time to give ethical matters thorough consideration when decisions are being made in the midst of a major crisis (Thomas et al., 2007). This is true also of social science research. While it is my firm belief that public engagement work by geographers can play an important role in recording, understanding and anticipating, the societal effects of planetary‐scale emergencies (Rose‐Redwood et al., 2020), the ethical effects of such work need to be carefully examined and frequently re‐examined.

Despite the relative absence of substantive debate on dignity in geographic research, teaching and public engagement, institutionalised ethical policies and protocols frequently evoke dignity as if its meaning is known and shared. Thus, through their institutional procedures as well as the social‐spatial relations they research, geographers already engage with dignity. Given the concept's fraught histories, however, there is a need to challenge taken‐for‐granted uses, and propose appropriate alternative—likely partial and contingent—notions of dignity (Nahman, 2008). As feminist and postcolonial scholars in anthropology, social work and human rights in particular have argued, dignity may be more usefully thought of as intersubjective, affective, relational and situated (Becker, 2021). In considering the utility of a reformulated approach to dignity for urban geography, Grossmann and Trubina argue that dignity is ‘deeply grounded in everyday human spatial sensibilities’ (Grossmann & Trubina, 2022, p. 2). However, there is a need for further empirical and theoretical explorations of what the effects are of geographers'—whether explicit or implicit—reliance on dignity.

In this paper, I have argued that public engagement with research involves entanglements with dignity as relational, problematic, contextual and partial. I have proposed that it can be a useful lens in planning and conducting engagement projects, particularly if considered in relation to specific interactions, and not as an inherent individual quality which the researcher can affirm, or take away. A discussion of dignity has relevance not only to managing public engagement with research ethically; it has implications for the wider conceptualisation and practice of public geography. A re‐thinking of dignity can be an important extension to geographers' ongoing engagement with care, positionality and reflexivity (Bourlessas, 2021; Hopkins, 2007; Rose, 1997).

One conclusion from the above account concerns the limited ethical guidance which researchers engaging with non‐academic publics currently receive. One of the challenges in ‘doing’ public engagement comes from the reliance on tacit knowledge for practicing it ethically, the risks associated with this, and the relative absence of spaces in which to discuss, scrutinise and learn from others (for a related argument on qualitative methods in geography, see Latham, 2020). In the case of my own institution, formal ethical approval was only required for the ‘research part’ of the project, even though such a rigid distinction seems artificial from within the messiness of engagement work (Craig, 2019). This approach, with its narrow focus on the material used in academic publications, seems relatively better‐suited to the protection of data (an important, but incomplete framing of participant dignity) than to the wider ethical and political consequences of the interactions between academics and different publics. The need to pay attention to the ethical dimensions of public geographies has been discussed before (Fuller & Askins, 2010). This need in turn opens up the complex discussion of the burdens which further institutional ethical approval processes would place on academic and professional staff. In the context of unsustainable yet growing workloads, it is difficult to argue for more procedures and paperwork. However, the systemic issues of workloads in the academy should be a reason for more, not fewer, meaningful engagements with questions of ethics. Critically reflecting on what geographers mean when we talk about dignity, can be an important part of these engagements.

## Data Availability

The data that support the findings of this study are available on request from the corresponding author. The data are not publicly available due to privacy or ethical restrictions.
